# Retractile spermatic cord hernia: An unusual case report

**DOI:** 10.1097/MD.0000000000045464

**Published:** 2025-11-14

**Authors:** Zhiwei Gu, Wei Liu, Bin Li, Zhenguo Qiao, Gang Wang, Yang Bai

**Affiliations:** a Department of Minimally Invasive Common Surgery, Suzhou Ninth People’s Hospital, Xuzhou Medical University Suzhou Bay Clinical College, Suzhou, Jiangsu Province, China; b Department of Gastroenterology, Suzhou Ninth People’s Hospital, Xuzhou Medical University Suzhou Bay Clinical College, Suzhou, Jiangsu Province, China.

**Keywords:** case report, hernia sac, inferior epigastric vessels, inguinal hernia, spermatic cord, total extraperitoneal laparoscopic inguinal hernia repair

## Abstract

**Rationale:**

Retractile spermatic cord hernia is an extremely rare type of inguinal hernia in which the herniated contents are located posterior to the spermatic cord, rather than passing anterior to it into the scrotum or medial thigh as in typical cases. To date, no case reports have described laparoscopic treatment of retractile spermatic cord hernia. This study presents a complete surgical case, highlighting the rarity of this condition and providing valuable reference for surgical decision-making in similar clinical scenarios.

**Patient concerns:**

A 59-year-old male presented with a 1-year history of left inguinal discomfort and swelling. He recently noticed a recurrent lump in the same region without systemic symptoms such as fever or gastrointestinal disturbance.

**Diagnoses:**

Ultrasonography confirmed the diagnosis of a left inguinal hernia. During surgery, a retractile hernia sac was found posterior to the spermatic cord, located near the left inferior epigastric vessels, and containing extraperitoneal fat and peritoneum.

**Interventions:**

The patient underwent total extraperitoneal laparoscopic hernia repair under general anesthesia. Dissection of the hernia sac was performed carefully, and a Bard 3DMax™ lightweight mesh was placed to reinforce the preperitoneal space.

**Outcomes:**

The surgery was completed successfully without intraoperative or postoperative complications. The patient recovered well, and no recurrence was observed during follow-up.

**Lessons:**

Retractile spermatic cord hernia is clinically rare and can be easily confused with common inguinal hernias, often being diagnosed only intraoperatively. Total extraperitoneal laparoscopic hernia repair provides a clear operative field and facilitates rapid postoperative recovery. Increased awareness of this hernia type and accurate intraoperative identification are crucial for preventing tissue injury and optimizing surgical decision-making.

## 1. Introduction

Retractile spermatic cord hernia is a rare yet clinically significant type of hernia. It is characterized by the downward protrusion of tissues, typically part of the intestine, through the inguinal region into the scrotum or inner thigh, with entry along the posterior aspect of the spermatic cord. Notably, the hallmark feature of this herniation is the reversal of the hernial sac’s path from the normal anatomical position of the spermatic cord, with the hernia sac located posteriorly.^[1–3]^ The pathogenesis of retractile spermatic cord hernia involves abnormalities in abdominal anatomical structures and the anatomical relationships of the spermatic cord. These anomalies may arise from defects in inguinal region anatomy, congenital developmental abnormalities, or dysfunction of muscles or peritoneum. Diagnosing and surgically treating retractile spermatic cord hernia pose certain challenges.^[4]^

This article presents a case of successful surgical repair of retractile spermatic cord hernia utilizing total extraperitoneal laparoscopic inguinal hernia repair.^[5–7]^ The aim is to share surgical experience and clinical insights, providing valuable references and inspiration for the treatment of similar conditions.

## 2. Case report

The patient, a 59-year-old male, presented to our hospital on March 26, 2024, with a complaint of discomfort and swelling in the left inguinal region persisting for over a year, accompanied by the repeated protrusion of a lump for the past month. He has a history of hypertension managed with oral medication. On admission, physical examination revealed a palpable lump approximately 4 cm × 4 cm × 3 cm in size protruding from the left inguinal region, without entering the scrotum. The lump was non-tender, smooth, with clear margins, and reducible on supine positioning. Examination findings included an external ring diameter increased by approximately 2 fingers and an impulse sensation upon coughing. Bilateral testes and epididymis examination showed no abnormalities, and the transillumination test of the scrotum was negative. Ultrasonography confirmed a left inguinal hernia. The patient was diagnosed with a left inguinal hernia and hypertension upon admission. Further investigations revealed no significant surgical contraindications.

**Figure 1.**
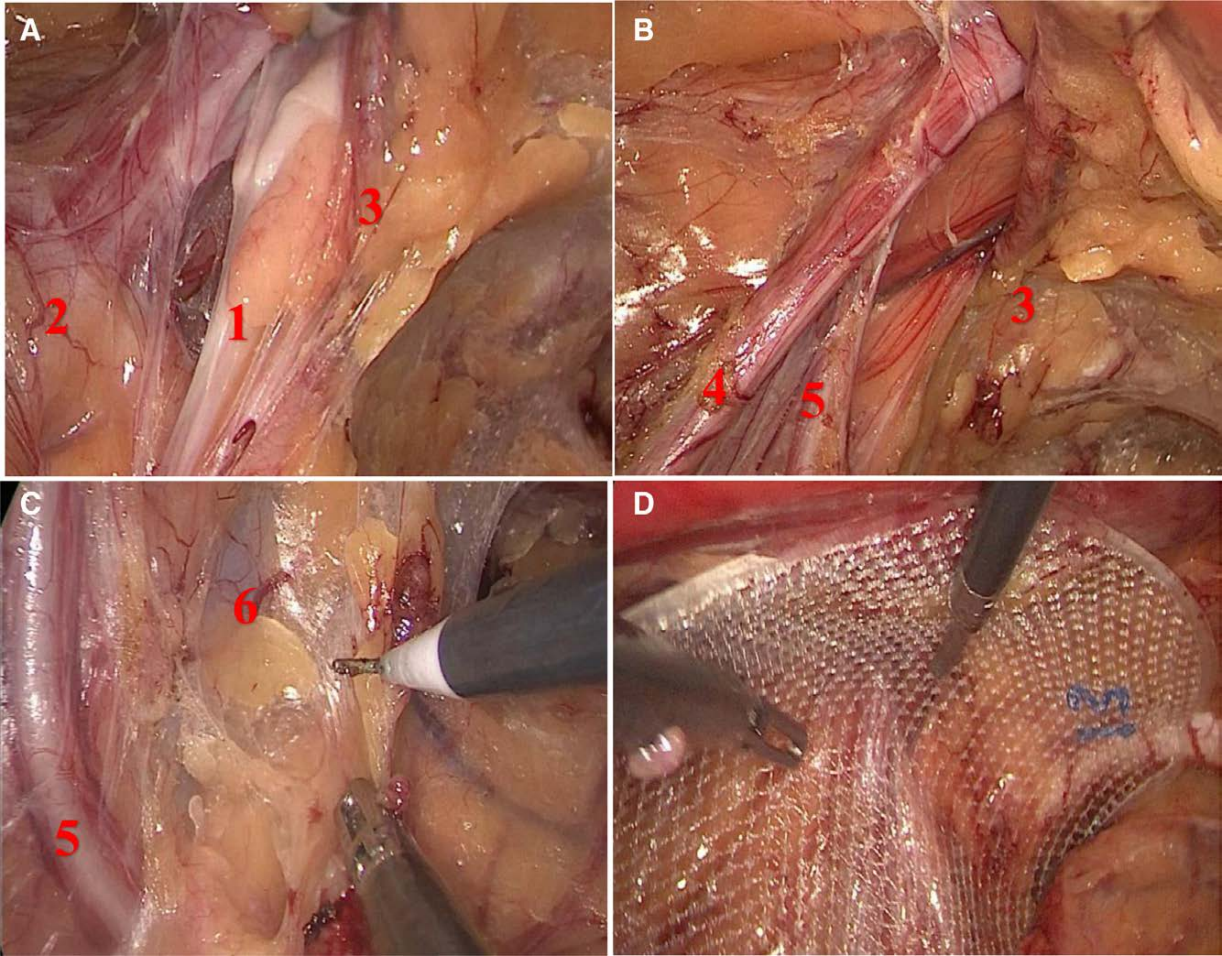
Pictures in this case. (A) Reduction of the hernia sac behind the spermatic cord in the inguinal region. (B) Full exposure of the abdominal wall defect. (C) The position relationship between the hernia sac below and the iliac vessels. (D) Placement of Bard 3Dmax lightweight mesh (hernia sac, peritoneal flap, inferior epigastric vessels, apermatic cord vessels, vas deferens, and iliac vessels).

On March 28, 2024, under general anesthesia, the patient underwent successful total extraperitoneal laparoscopic inguinal hernia repair. During the intraoperative assessment, the left hernia sac, measuring approximately 4 cm × 4 cm × 3 cm, was observed positioned behind the spermatic cord. It protruded closely adjacent to the outer side of the left inferior epigastric vessels, containing extraperitoneal fat tissue and peritoneum (Fig. 1A, B). The sac was meticulously dissected, reduced, and the spermatic cord was freed from the peritoneum (Fig. 1C). Subsequently, a Bard 3Dmax lightweight mesh (10.3 cm × 15.7 cm) was placed after ensuring the integrity of the preperitoneal space (Fig. 1D). The surgery was completed successfully without intraoperative complications. The patient experienced minimal postoperative pain, recovered well, and was discharged on the second postoperative day. No immediate postoperative complications were observed.

During follow-up at 1 and 3 months, the patient remained asymptomatic with no signs of hernia recurrence. Physical examination and ultrasonography confirmed the absence of complications, and the patient reported satisfactory functional recovery.

## 3. Discussion

This case of retractile spermatic cord hernia offers several valuable insights into the presentation, diagnosis, and surgical management of this rare condition.

### 3.1. Patient presentation and symptom characteristics

The patient presented with prolonged discomfort and aching in the left inguinal region, which was later accompanied by a reducible lump. This presentation diverges from the typical symptoms associated with anterior inguinal hernias. The posterior positioning of the hernia sac relative to the spermatic cord may contribute to these atypical symptoms. Because the spermatic cord partially obscures the hernia sac, its initial protrusion through the internal inguinal ring is less likely, leading to a delayed or less obvious presentation. As intra-abdominal pressure increases and abdominal wall tension decreases, the hernia sac gradually protrudes, and the inguinal lump becomes more noticeable but remains reducible. This pattern suggests that patients with posterior inguinal hernias may primarily experience persistent discomfort in the inguinal region for an extended period before a palpable lump emerges. Furthermore, even when a lump does appear, it may not be substantial in size. This atypical symptomatology highlights the need for a high index of suspicion and careful clinical assessment, especially in cases where traditional signs of inguinal hernia are absent. Understanding these unique presentation characteristics is crucial for early diagnosis and appropriate management.

### 3.2. Key surgical procedures

The surgical approach for retractile spermatic cord hernia involves several critical steps, including detachment, reduction, and peritonealization of the hernia sac. These maneuvers are generally more straightforward compared to those required for anterior inguinal hernias, largely due to the different anatomical relationships involved. The peritonealization of the spermatic cord, for instance, is more akin to the technique used in direct inguinal hernia repair, making it relatively less complex. However, the unique anatomy of retractile spermatic cord hernia necessitates special precautions to avoid injury to adjacent structures. The proximity of the hernia sac neck to critical anatomical landmarks such as the left inferior epigastric vessels and the vas deferens on the right side demands meticulous dissection and precise reduction techniques (Fig. 1A, B). Additionally, the iliac vessels’ close proximity during the downward dissection requires vigilant protection to prevent vascular injury (Fig. 1C). These anatomical considerations underscore the importance of careful and deliberate surgical planning and execution to minimize the risk of complications and ensure successful outcomes.

### 3.3. Advantages of laparoscopic surgery

Laparoscopic techniques offer distinct advantages in the management of posterior inguinal hernias, particularly retractile spermatic cord hernias. The enhanced visualization provided by laparoscopy allows for a clearer delineation of the hernia and its relationship to surrounding structures such as the inferior epigastric vessels, vas deferens, spermatic cord vessels, and iliac vessels. This improved visualization facilitates precise dissection and minimizes the risk of inadvertent injury to these critical structures. The minimally invasive nature of laparoscopic surgery also contributes to reduced postoperative pain, shorter hospital stays, faster recovery times, and lower rates of postoperative complications compared to open surgical techniques. Moreover, the ability to perform a tension-free repair using mesh, such as the Bard 3Dmax lightweight mesh, further enhances the durability and success of the procedure by reducing the risk of hernia recurrence.

### 3.4. Clinical implications and recommendations

Given the rarity of retractile spermatic cord hernia,^[8]^ each reported case provides a valuable opportunity to enhance our understanding of its presentation, diagnosis, and management. The insights gained from this case highlight the importance of individualized treatment planning based on the specific anatomical and clinical characteristics of each patient. Surgeons should be prepared to modify their approach based on intraoperative findings and the unique challenges presented by this rare type of hernia. Additionally, meticulous surgical technique, including careful dissection and protection of vital structures, is paramount to reducing complications and achieving optimal outcomes. Regular postoperative follow-up is essential to monitor for recurrence and manage any delayed complications. As more cases are reported and our experience grows, we will be better positioned to refine our surgical techniques and improve patient outcomes further.

This study has several limitations. First, as a single case report, the findings may not be generalizable to all patients with retractile spermatic cord hernia. Second, the lack of long-term follow-up limits the evaluation of recurrence and late postoperative complications. Third, the rarity of this condition precluded comparative analysis with alternative surgical techniques. Finally, the intraoperative findings were specific to this patient and may vary in others with different anatomical features. Despite these limitations, this report provides valuable insights for clinicians managing this uncommon hernia type.

## 4. Conclusion

In conclusion, this case underscores the need for heightened awareness of atypical hernia presentations and the benefits of laparoscopic techniques in managing rare inguinal hernias. By tailoring treatment strategies to the individual patient and employing meticulous surgical practices, we can enhance treatment efficacy and improve patient quality of life. Further research and case accumulation are needed to establish standardized protocols and optimize the care of patients with retractile spermatic cord hernia.

## Acknowledgments

We thank the patient for agreeing to let us report on this case report.

## Author contributions

**Writing – original draft:** Zhiwei Gu, Wei Liu, Bin Li, Zhenguo Qiao, Gang Wang, Yang Bai.

**Writing – review & editing:** Zhiwei Gu, Wei Liu, Bin Li, Zhenguo Qiao, Gang Wang, Yang Bai.
